# Partial versus early full weight bearing after uncemented total hip arthroplasty: a meta-analysis

**DOI:** 10.1186/s13018-017-0527-x

**Published:** 2017-02-17

**Authors:** Peng Tian, Zhi-jun Li, Gui-Jun Xu, Xiao-lei Sun, Xin-long Ma

**Affiliations:** 1 Department of Orthopedics, Tianjin Hospital, No. 406, Jiefang Nan Road, Tianjin, 300211 People’s Republic of China; 2 Department of Orthopedics, Tianjin Medical University General Hospital, Tianjin, 300052 People’s Republic of China

**Keywords:** Hip, Arthroplasty, Uncemented, Weight bearing, Meta-analysis

## Abstract

**Background:**

This meta-analysis aimed to investigate the efficacy and safety of partial weight bearing (PWB) versus early full weight bearing (FWB) after uncemented total hip arthroplasty (THA).

**Methods:**

We conducted a search in PubMed, EMBASE, The Cochrane Library, and Web of Science for randomized controlled trials (RCTs) and non-RCTs comparing PWB and early FWB after uncemented THA. Two authors conducted the selection of studies, data extraction, and assessment of risk of bias independently. A pooled meta-analysis was performed using the RevMan 5.3 software.

**Results:**

Six RCTs and three non-RCTs met the inclusion criteria. The meta-analysis indicated that compared with PWB, the FWB group showed greater femoral subsidence at 3-month follow-up (MD = −0.12, 95% CI −0.22 to −0.01, *P =* 0.03). There were no significant differences in the hip Harris score at 1-year and 2-year follow-up (MD = 1.54, 95% CI −0.83 to 3.90, *P =* 0.20; MD = 0.08, 95% CI −1.19 to 1.34, *P =* 0.90, respectively), in femoral subsidence at 2-year follow-up and at two additional years of follow-up (MD = −0.03, 95% CI −0.31 to 0.25, *P =* 0.84; (MD = −0.02, 95% CI −0.37 to 0.33, *P =* 0.91, respectively). There were no significant differences in the incidences of bone ingrowth fixation, spot welds, and radiolucent lines.

**Conclusions:**

This meta-analysis shows that early FWB in patients with uncemented THA could be safe and could not increase the incidence of postoperative complications.

## Background

As society ages, the incidences of osteoarthritis of the hip and femoral neck fracture increase year by year [1, 2]. Total hip arthroplasty (THA) has long been recognized as the most effective surgical method in the treatment of hip diseases. The optimal method of fixation for THA, particularly fixation with or without cement, remains controversial [3].

Although cemented THA could be suitable for elderly patients, and the prognosis would be relatively better [4, 5], increasing early loosening rates of cemented THA prostheses have been reported [6]. Younger patients who underwent cemented THA have exhibited higher revision rates due to more exercise [7]. Thus, cemented prostheses could be restricted, and uncemented prostheses would be widely used in clinics [8].

Some studies have proposed that the time of partial weight bearing (PWB) of patients with uncemented THA should last for 6 to 12 weeks [9, 10]. However, others have suggested that early postoperative full weight bearing would not affect the stability of the prosthesis but could shorten the rehabilitation training and prevent disuse osteoporosis, bedsores, hypostatic pneumonia, and other complications, and hence, it was highly recommended that patients who accepted uncemented THA should perform early postoperative full weight bearing (FWB) activities [10, 11]. The initial stability and bone ingrowth of the uncemented prosthesis would be affected by the design of the prosthesis, which could not be ignored in the choice between postoperative PWB and FWB [11].

Although FWB and PWB are both used in THA, controversies over their efficacy and safety still exist. The purpose of this meta-analysis is to compare the effects of FWB versus PWB in patients with uncemented THA to provide a reference for THA.

## Methods

### Search strategy

To identify all available studies, no languages were restricted. According to the guidelines of the Cochrane Collaboration, we first conducted an electronic search of major databases including PubMed, EMBASE, The Cochrane Library, and Web of Science using the following terms: “Hip arthroplasty”, “Uncemented,” and “Weight bearing” with publication dates from January 1966 to September 2016. We then manually searched the reference lists of all included studies, relevant books, review articles, and meeting proceedings to identify trials that might have been missed in the electronic search. To gain precise data, two reviewers were scheduled to independently conduct electronic and manual searches based on the title, abstract, and full-text articles when necessary. Any disagreements were resolved through discussion. We carefully reviewed studies published by the same team to ensure that the same results were not included twice.

### Selection criteria

We included trials following these characteristics: (1) comparative studies (randomized controlled trials (RCTs) or non-RCTs); (2) comparison of PWB and FWB after uncemented THA; and (3) full-text articles with detailed information. Exclusion criteria were articles for which we were unable to obtain the full text and papers lacking available information.

### Quality assessment

After we identified these eligible studies, quality assessment was conducted. According to whether the study is a randomized or non-randomized trial, the index for non-randomized studies (MINORS) form was used to assess retrospective controlled trials [12], while a modification of the generic evaluation tool used by the Cochrane Bone, Joint and Muscle Trauma Group was used for randomized trials. The methodological quality of each trial was scored from 0 to 24. To assess the methodological quality of RCTs, we applied the Cochrane Collaboration’s tool for assessing the risk of bias, which includes the following key domains: adequate sequence generation, allocation concealment, blinding, incomplete outcome data, selective reporting, and other bias. Disagreements were resolved by discussion to reach a consensus or by consultation with the senior reviewer.

### Data extraction

Two authors independently extracted data from the eligible studies according to a predefined plan, including the following information: study design, patient demographics, interventions, outcomes, and follow-up duration for each treatment group and any other outcomes mentioned in individual studies using a standardized review form. Attempts were made to contact authors for supplementary information when the reported data were inadequate or unclear. All data were checked for consistency, missing values, and validity.

### Data analysis and statistical methods

RevMan 5.3 for Windows (Cochrane Collaboration, Oxford, UK) was used to conduct the statistical analyses. We estimated the heterogeneity using a standard chi-square test (significant at *P* values less than 0.05 and *I* ^2^ values greater than 50%) [13]. When significant heterogeneity existed, pooled data were analyzed using a random-effects model [14]. Otherwise, a fixed-effects model was used for the analysis. Publication bias was evaluated visually using funnel plots. It was considered asymmetric when the *P* value of the slope coefficient was less than 0.05. Sensitivity analyses were undertaken in clinical remission and response. Risk difference (RD) and 95% confidence intervals (CI) were calculated for dichotomous outcomes, while mean difference (MD) and 95% CI were calculated for continuous outcomes.

## Results

### Search results

**Fig. 1:**
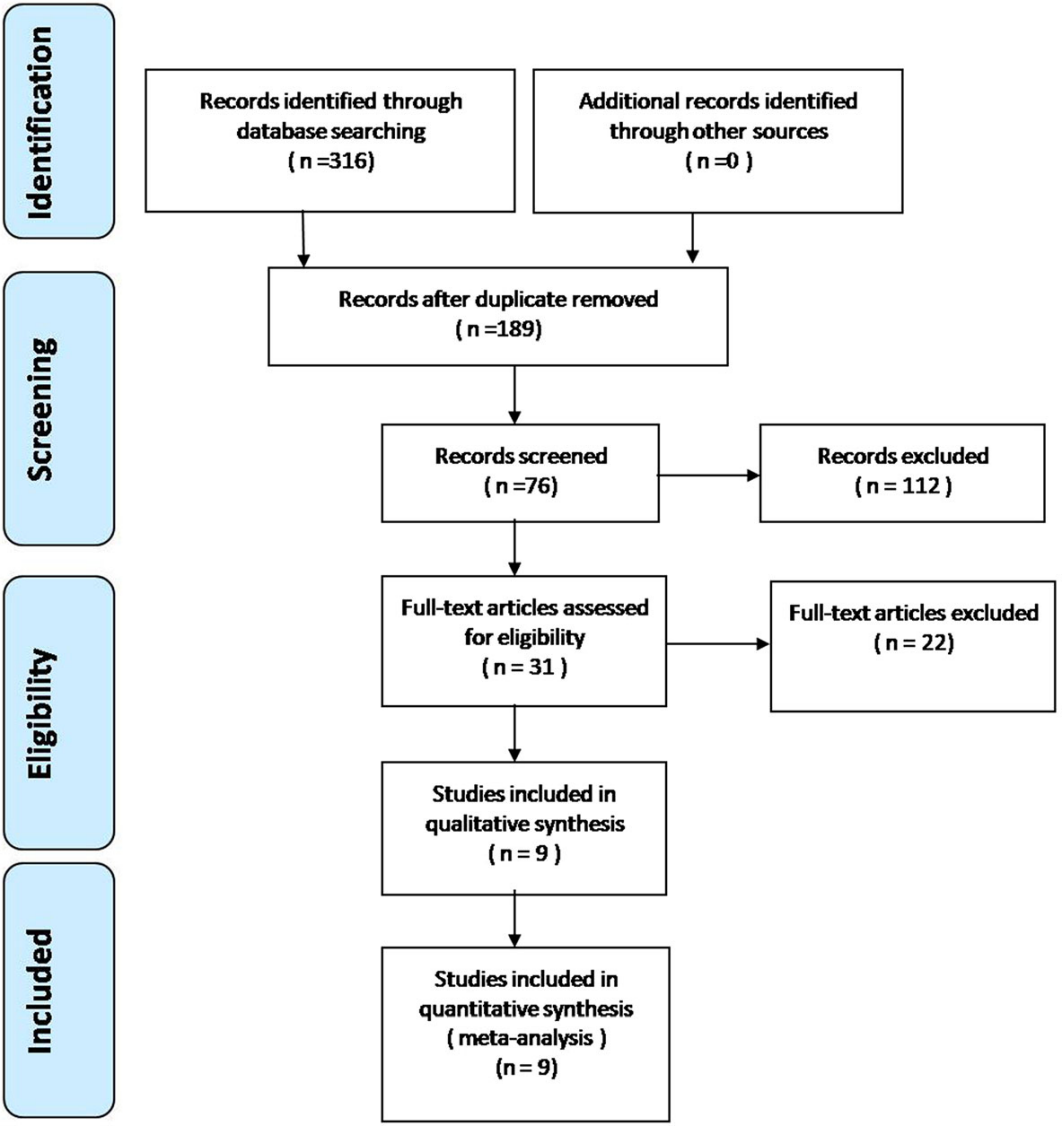
Flow chart of the study selection and inclusion process

A total of 316 studies were identified as potentially relevant literature reports. By scanning the titles and abstracts, 307 reports were excluded based on the eligibility criteria. No additional studies were obtained after the reference review. Ultimately, six RCTs and three non-RCTs were eligible for data extraction and meta-analysis [10, 11, 15–21]. The search process is shown in Fig. 1.

### Study characteristics

The characteristics of the nine included studies are shown in Table 1. Statistically similar baseline characteristics were observed between both groups. All studies had small sample sizes, from 20 to 100 hips.

**Table 1:** Cohort characteristics

Studies	Design	Hips (FWB/PWB)	Mean age (FWB/PWB)	Male patients (FWB/PWB)	Body weight (FWB/PWB)	Intervention		Follow-up (year)
						FWB	PWB	
Rao et al. 1998 [11]	CS	28/28	52/55	12/12	74/75	PO 1 day	10% BW*6 weeks	2
Kishida et al. 2001 [10]	RCT	19/18	52/51	11/12	59/58	PO 1 day	FWB*3–6 weeks	5
Woolson and Adler 2002 [21]	CS	25/25	65/54	14/16	80/86	PO immediately	<50 lb*4 weeks	5
Chan et al. 2003 [15]	CS	29/29	50/51	17/17	61/58	PO immediately	Protected WB*6 weeks	2
Bodén and Adolphson 2004 [16]	RCT	10/10	54/55	NS	NS	PO	10% BW	2
Unver et al. 2004 [19]	RCT	24/27	50/49	NS	70/67	PO 2 days	PWB*6–8 weeks	1
Thien et al. 2007 [20]	RCT	19/19	53/54	NS	82/76	PO 1 day	<30 kg*6 weeks	1
Ström et al. 2007 [18]	RCT	21/21	55/56	12/10	80/79	PO immediately	15 kg*3 months	2
Markmiller et al. 2011 [17]	RCT	50/50	61/61	19/22	80/76	PO immediately	15 kg*6 weeks	2

*FWB* full weight bearing, *PWB* partial weight bearing, *CS* cohort study, *RCT* randomized controlled trial, *NS* not state, *PO* post-operative, *BW* body weight

### Risk of bias assessment

**Fig. 2:**
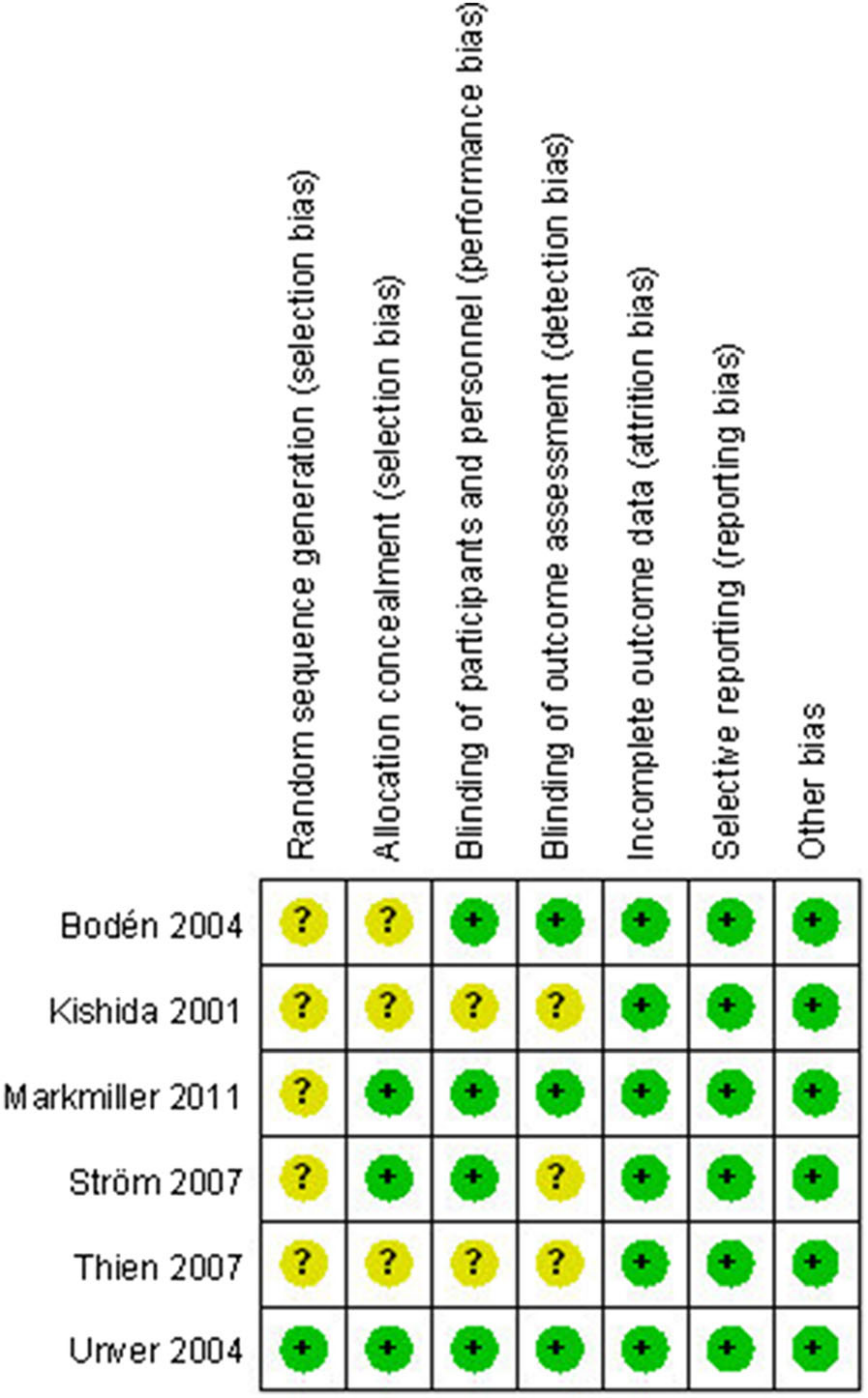
The summary of bias risk of randomized controlled trials

The RCT quality was assessed based on the Cochrane Handbook for Systematic Review of Interventions (Fig. 2). One RCT clearly stated the methodology of randomization, while the others did not provide a methodology of randomization. The concealment of allocation was adequate in three RCTs. Blinding of the assessor and participants was provided in four RCTs. No unclear bias due to incomplete outcome data or selective outcomes was reported. For the non-RCTs, the MINORS scores were 16–18 for the retrospectively controlled trials. The methodological quality assessment is illustrated in Table 2.

**Table 2:** Quality assessment for non-randomized trials

Quality assessment for non-randomized trials	Rao et al. 1998 [11]	Woolson and Adler 2002 [21]	Chan et al. 2003 [15]
A clearly stated aim	2	2	2
Inclusion of consecutive patients	1	2	2
Prospective data collection	2	2	2
Endpoints appropriate to the aim of the study	1	1	1
Unbiased assessment of the study endpoint	0	0	0
A follow-up period appropriate to the aims of study	2	2	2
Less than 5% loss to follow-up	2	2	2
Prospective calculation of the sample size	0	0	0
An adequate control group	2	1	2
Contemporary groups	1	0	1
Baseline equivalence of groups	2	2	2
Adequate statistical analyses	2	2	2
Total score	17	16	18

### Outcomes of meta-analysis

#### Harris score

One included study demonstrated that during the first 3 months after uncemented THA, the Harris scores of hip joints in the FWB group were higher than in the PWB group [19].

Postoperative Harris scores at 1-year follow-up were reported in two included studies. No significant heterogeneity was found, and a fixed-effects model was applied (*I* ^2^ = 17%, *P =* 0.27). The postoperative Harris score at 1-year follow-up in the FWB group was not significantly higher than in the PWB group (MD = 1.54, 95% CI −0.83 to 3.90, *P =* 0.20; Table 3).

**Table 3:** Meta-analysis results

Outcome	Studies	Groups (FWB/PWB)	Overall effect			Heterogeneity	
			Effect estimate	95% CI	*P* value	*I* ^2^(%)	*P* value
Harris score							
1-year follow-up	2	53/56	1.54	−0.83, 3.90	0.20	17	0.27
2-year follow-up	4	92/92	0.08	−1.19, 1.34	0.90	29	0.24
Femoral subsidence							
3-month follow-up	3	87/85	−0.12	−0.22, −0.01	0.03	0	0.90
2-year follow-up	5	128/128	−0.03	−0.31, 0.25	0.84	3	0.38
≥2-year follow-up	6	146/143	−0.02	−0.37, 0.33	0.91	74	0.002
Bone growth fixation	6	160/159	0.00	−0.03, 0.03	1.00	0	1.00
Spot welds	3	63/63	0.08	−0.05, 0.21	0.24	0	0.39
Radiolucent lines	5	133/132	−0.00	−0.07, 0.06	0.96	0	0.99
Prosthetic loosening	8	206/208	−0.00	−0.03, 0.02	0.74	0	1.00

Postoperative Harris scores at 2-year follow-up were reported in four included studies. No significant heterogeneity was found, and a fixed-effects model was applied (*I* ^2^ = 29%, *P =* 0.24). The postoperative Harris score at 2-year follow-up in the FWB group was not significantly higher than in the PWB group (MD = 0.08, 95% CI −1.19 to 1.34, *P =* 0.90; Table 3).

#### Femoral subsidence

Femoral component subsidence was defined as a change of more than 4 mm [22]. Femoral subsidences at 3-month follow-up were reported in three included studies. No significant heterogeneity was found, and a fixed-effects model was applied (*I* ^2^ = 0%, *P =* 0.90). Femoral subsidence at 3-month follow-up in the FWB group was significantly higher than in the PWB group (MD = −0.12, 95% CI −0.22 to −0.01, *P =* 0.03; Table 3).

Femoral subsidences at 2-year follow-up were reported in five included studies. No significant heterogeneity was found, and a fixed-effects model was applied (*I* ^2^ = 3%, *P =* 0.38). Femoral subsidence at 2-year follow-up in the FWB group was not significantly higher than in the PWB group (MD = −0.03, 95% CI −0.31 to 0.25, *P =* 0.84; Table 3).

Femoral subsidences at two more years follow-up were reported in six included studies. Significant heterogeneity was found, and a random model was applied (*I* ^2^ = 74%, *P =* 0.002). Femoral subsidence at two more years follow-up in the FWB group was not significantly higher than in the PWB group (MD = −0.02, 95% CI −0.37 to 0.33, *P =* 0.91; Table 3).

#### Bone ingrowth fixation

The fixation of the femoral components was assessed radiographically according to the Engh criteria [23]. Bone ingrowth fixation was reported in six included studies (160/161 and 159/160, respectively). No significant heterogeneity was found, and a fixed-effects model was applied (*I* ^2^ = 0%, *P =* 1.00). Bone growth fixation in the FWB group was not significantly higher than in the PWB group (RD = 0.00, 95% CI −0.03 to 0.03, *P =* 1.00; Table 3). The incidences of bone ingrowth fixation in FWB and PWB groups are 160/161 and 159/160, respectively.

#### Spot welds

Spot weld was defined as new cancellous bone formation between the implant and the endosteal surface of the femur seen on follow-up radiographs [23]. Three studies reported the incidence of spot welds (41/63 and 36/63, respectively). There was no significant heterogeneity (*I* ^2^ = 0%, *P =* 0.39); therefore, a fixed-effects model was applied. Pooling the results demonstrated that the incidence of spot welds in the FWB group was not significantly lower than in the PWB group (RD = 0.08, 95% CI −0.05 to 0.21, *P =* 0.24; Table 3).

#### Radiolucent lines

Radiolucent lines were parallel with and in close proximity to the implant and was associated with a thin radiopaque layer of bone paralleling the line [15]. Radiolucent lines were reported in five of the studies (10/133 and 10/132, respectively). No significant heterogeneity was found, and a fixed-effects model was used (*I* ^2^ = 0%, *P =* 0.99). The incidence of radiolucent lines in the FWB group was not significantly higher than in the PWB group (RD = −0.00, 95% CI −0.07 to 0.06, *P =* 0.96; Table 3).

#### Prosthetic loosening

Prosthetic loosening was defined as a migration or breakage of the prosthesis [24]. Prosthetic loosening was reported in eight of the studies (0/206 and 1/208, respectively). No significant heterogeneity was found, and a fixed-model was used (*I* ^2^ = 0%, *P =* 1.00). The incidence of prosthetic loosening in the FWB group was not significantly higher than in the PWB group (RD = −0.00, 95% CI −0.03 to 0.02, *P =* 0.74; Table 3).

## Discussion

Uncemented THA is widely used in the treatment of femoral neck fractures and other hip diseases, but the choice of postoperative weight-bearing timing remains controversial. Some scholars believed that early FWB could increase femoral stem subsidence and aggravate poor initial stability, leading to a high rate of hip revision [8]. Therefore, some scholars recommended that the time of PWB of patient with uncemented THA should last for 6 to 12 weeks [9, 10]. The current meta-analysis provides evidence-based support to allow immediate FWB after uncemented THA. There was no correlation between the degree of femoral stem subsidence and the actual weight of the hip joint after the uncemented THA. FWB did not increase the short-term or long-term subsidence of femoral stem prostheses [18] and did reduce the risk of deep vein thrombosis [25]. Early FWB and active rehabilitation could be recommended for the uncemented CLS stem [18]. Therefore, it is necessary to recommend early FWB after uncemented THA.

This meta-analysis showed no significant difference in Harris scores between the two groups during the first and second years after uncemented THA (MD = 1.54, *P =* 0.20; MD = 0.08, *P =* 0.90, respectively). However, one study demonstrated that during the first 3 months after uncemented THA, the Harris scores of hip joints in the FWB group were higher than in the PWB group [19]. Although there was a significant difference in Harris scores between the two groups during the early stages, as the patients in the PWB group began to complete weight-bearing and active functional exercise, in the late stages, there was no advantage in the Harris scores of patients in the FWB group over patients in the PWB group. Postoperative FWB could promote the early recovery of the hip joint in patients with uncemented THA, which resulted in high social and economic value [21].

The initial stability of the uncemented femoral stem prosthesis depends on the mechanical match between the femoral stem prosthesis and the bone marrow cavity; the long-term stability of the uncemented femoral stem prosthesis is determined by mechanical matching and bone ingrowth [17]. If the femoral stem prosthesis does not achieve a full match when placed in the femoral medullary cavity, the femoral stem prosthesis would descend along the medullary cavity in the late weight-bearing activities until a tight matching occurred [11].

This meta-analysis showed that when patients with uncemented THA were followed up at 3 months, the femoral subsidences of patients with postoperative FWB were significantly higher than in patients with postoperative PWB (MD = −0.12, *P =* 0.03). However, there was no significant difference in femoral subsidences in the long-term follow-up of femoral stem prostheses between the two groups (MD = −0.03, *P =* 0.84 at 2-year follow-up; MD = −0.02, *P =* 0.91 at two more years follow-up, respectively). In the first postoperative 3 months, the reason for the lower femoral subsidences of patients with uncemented THA who underwent PWB was considered to be that when the prosthesis and the medullary cavity did not achieve the best matching, if the weight bearing increased gradually, the femoral stem subsidence began to catch up until fully matching the bone marrow cavity, until two or more years after surgery, when the femoral subsidences of the two groups would tend to be consistent. In other words, PWB could delay femoral stem subsidence, which did not provide long-term stability; and the delayed subsidence due to PWB would be offset before the prosthesis could reach long-term stability.

The patients with uncemented THA would perform PWB, which could cause an increase in the weight bearing of the contralateral hip joint. Limiting the weight bearing of the hip joint for 6 weeks could lead to muscle atrophy and bone loss around the hip joint, which would affect the recovery of hip function [19].

The inclusion criteria of this study were strictly controlled, and there was no significant heterogeneity among the outcomes. The results of the meta-analysis were reliable. Several potential limitations must be recognized in our meta-analysis: (1) the number of RCTs was limited, and partial cohort studies were included; (2) the sample size of some studies was small, and there may be publication bias; (3) postoperative rehabilitation methods as interventions may not be able to implement strict blindness; and (4) follow-up time was limited, as most outcome measures were followed up at 2 years after THA. Due to the above defects and deficiencies, the pooled estimates should be explained with caution.

## Conclusions

This meta-analysis shows that early FWB in patients with uncemented THA could be safe and could not increase the incidence of postoperative complications.

## Abbreviations

CI: Confidence intervals
FWB: Full weight bearing
MD: Mean difference
MINORS: Methodological Index for Non-randomized Studies
PWB: Partial weight bearing
RCT: Randomized controlled trial
RD: Risk difference
THA: Total hip arthroplasty
